# Long non-coding RNA long intergenic non-coding 00641 mediates cell progression with stimulating cisplatin-resistance in osteosarcoma cells via microRNA-320d/myeloid cell leukemia-1 axis

**DOI:** 10.1080/21655979.2022.2045090

**Published:** 2022-03-10

**Authors:** JinShan Tang, ZiQiang Zhu, Suwei Dong, YunQing Wang, JianQang Wang, HongLiang Chen, Gang Duan

**Affiliations:** a Department of Orthopedic, The Second People’s Hospital of Huai’an, Huai’an City, Jiangsu Province, China; b Department of Orthopedic, The Affiliated Huai’an Hospital of Xuzhou Medical University, Huai’an City, Jiangsu Province, China; c Department of Orthopedic, General Hospital of Xuzhou Mining Group, Xuzhou City, Jiangsu Province, China; d Department of Orthopedic, The Second Affiliated Hospital of Xuzhou Medical University, Xuzhou City, Jiangsu Province, China; e Department of Orthopedic, Affiliated Hospital of Xuzhou Medical University, Xuzhou City, Jiangsu Province, China

**Keywords:** Long intergenic non-coding 00641, osteosarcoma, cisplatin-resistance, proliferation, apoptosis

## Abstract

As a staple chemotherapy medicine, cisplatin (DDP) is extensively applied in cancer patients, but its drug resistance is limited. Numerous studies have elucidated that long non-coding RNA (lncRNA) performs as a pivotal agent in osteosarcoma (OS). Nevertheless, lncRNA long intergenic non-coding 00641 (LINC00641)’s functions in DDP resistance for OS remain obscure. The purpose of this study was to investigate the effect and mechanism of LINC00641 on drug resistance of OS. The tissues of both clinical cancer patients and the normal control were gathered. Detection of LINC00641, microRNA-320d (miR-320d) and myeloid cell leukemia-1 (MCL1) was conducted. After the selection of OS cell lines, the detection of cell advancement was applied. Series of experiments were conducted to verify the interaction of LINC00641, miR-320d and MCL1. Xenografted tumor model *in vivo* was utilized to determine the function of LINC00641. The data displayed, LINC00641 was prominently elevated in OS tissues and cells, especially in DDP-resistant tumors and cell lines. Knock-down LINC00641 was able to attenuate progression of DDP-resistant OS cells thus dampening their drug resistance toward DDP. Moreover, knock-downing LINC00641 gene was also able to manifest antagonism toward DDP-resistance *in vivo*. On the grounds of bioinformatics prediction, a direct binding of LINC00641 with miR-320d existed, whose target was MCL1. Meanwhile, LINC00641 modulated MCL1 via targeting miR-320d. Additionally, repressive LINC00641 blocked MCL1 via emulative interaction with miR-320d, thus expediting DDP-sensitivity of OS cells. All in all, it is found that LINC00641 is available to escalate drug resistance of DDP-resistant OS cells via mediation of miR-320d/MCL1 axis.

## Graphical abstract

**Figure:**
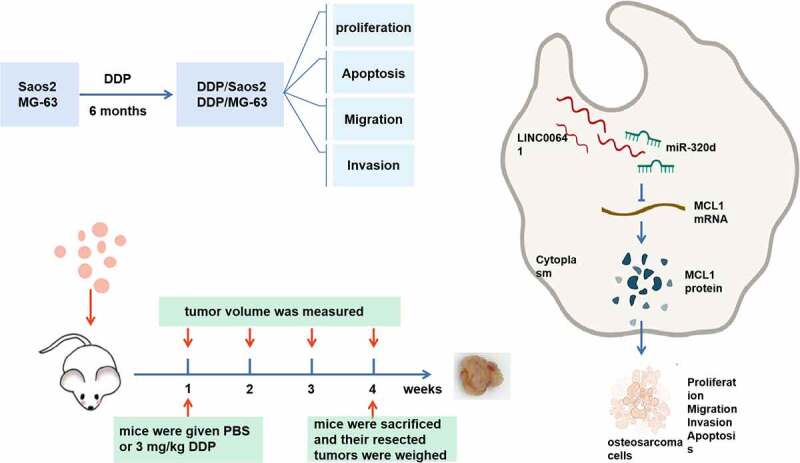

## 1 Introduction

Osteosarcoma (OS) is the most dominating malignant bone cancer among children and adolescents [1]. With the progress of radiotherapy, adjuvant chemotherapy and surgery (whose scope of tumor resection is more far-ranging), the global survival rate of most OS patients has remained ascending notably [2]. The universal treatment for OS patients is surgery combined with diverse medicine chemotherapy, like methotrexate, doxorubicin, cisplatin (DDP) and ifosfamide [3]. Even though the combination chemotherapy has achieved elevated survival rate, unfortunately, OS is a relatively drug-resistant disease, and the incidence rate of local recurrence and distant metastasis of OS patients after operation still stays augmented [3]. For the sake of exploring specific biomarkers and therapeutic targets, it is fairly indispensable to learn about the molecular mechanism of OS.

As a kind of non-coding RNA, long non-coding RNAs (lncRNAs) participate in miscellaneous physiological and pathological functions like cell progression, metabolism, differentiation ^[4–7]^. LncRNAs perform as a crucial agent in the process of OS. For instance, LncRNA MRPL23-AS1 activates Wnt/β-catenin signaling pathway by repressing microRNA (miR)-30b and upregulating myosin heavy chain 9 (MYH9) to promote the progress of OS and cancerization [8]; CRISPR/cas9-mediated overexpression of lncRNA SRY-box transcription factor 21 antisense divergent transcript 1 modulates the proliferation of OS by increasing the expression of mechanistic target of rapamycin kinase and Kruppel like factor 4 [9]. lncRNA OIP5-AS1 induces LPAATβ/PI3K/AKT/mTOR signaling pathway via sponging miR-340-5p, which contributes to DDP-resistance in OS [10]. LncRNA OIP5-AS1 modulates adriamycin-resistance via the mediation of miR-137-3p/PTN axis in OS [11]. LncRNA LINC00641 is located on chromosome 14q11.2, and it frequently descends under normal conditions [12]. Latest studies have clarified that LINC00641 is implicated in the tumorigenicity of bladder cancer, non-small cell lung cancer (NSCLC) and acute myeloid leukemia [^13–15^]. Nevertheless, the biological function of LINC00641 in OS and its resistance toward DDP have still stayed unclear.

MicroRNAs (miRNAs), a type of endogenous non-coding small RNA, demonstrate mediation of gene in the post-transcription level. A great many cellular pathways and functions, involving cell metabolism, differentiation and apoptosis, are under the modulation of miRNAs, hence their disorder will generate numerous human diseases [16]. An ascending number of studies have uncovered miRNAs take a pivotal effect in chemotherapy-resistance of cancer, and have verified that miRNAs have underlying effect in determining drug sensitivity or drug resistance [17]. With its abnormal manifestation in diverse kinds of tumors, it has been testified that miR-320d is momentous in NSCLC [18], cardiac adenocarcinoma [19]. Yet its function in DDP resistance of OS cells has remained still undefined.

Myeloid cell leukemia −1 (MCL1) belongs to Bcl-2 family [20]. A great many literatures and studies have expounded that MCL1 is rather vital in the survival and death of miscellaneous cells [21,22]. It has been indicated that miR-26a reverses multidrug resistance of OS via targeting MCL1 [23]. Yet it has remained unknown whether LINC00641 signifies the mediation of MCL1 in OS.

This study was to figure out how LINC00641 manifests in DDP-resistant tissues and cell lines in OS, and to explore the influence of LINC00641 on DDP-resistance in OS patients *in vivo* and *in vitro*. Apart from that, the author also discussed the latent molecular mechanism of LINC00641 in DDP-resistance. It was hypothesized that LINC00641 regulated cell proliferation, invasion and apoptosis and promoted DDP resistance of OS cells through miR-320d/MCL1 axis. This study was inclined to be conducive to providing an underlying therapeutic method for OS.

## 2 Materials and methods

### 2.1 Patients and specimens

The study gained authorization from the Ethics Committee of Huai’an Second People’s Hospital, and collection of written informed consent was from both Huai’an Second People’s Hospital and all patients. After the resection operation of patients in Huai’an Second People’s Hospital, both OS tissues and para-cancerous normal tissues were gathered from 58 OS patients. All cancer tissue specimens were diagnosed as OS via pathological examination. The quick refrigeration and preservation in liquid nitrogen of all fresh samples were exerted until further experiments. Then, 58 patients with OS, on the grounds of their sensitivity to chemotherapy medicine, were assigned into two groups. One was the chemotherapy sensitive (the tumor was relieved after six cycles of chemotherapy, n = 24), the other was the chemotherapy resistant (the tumor emerged stability or progression after six cycles of chemotherapy, n = 34). Additionally, 58 patients were assigned into two groups in line with LINC00641 expression, and calculation of the total survival period of all participants was in different stages (0, 20, 40, 60 months) after DDP therapy.

### 2.2 Cell culture and transfection

**Figure 1.**
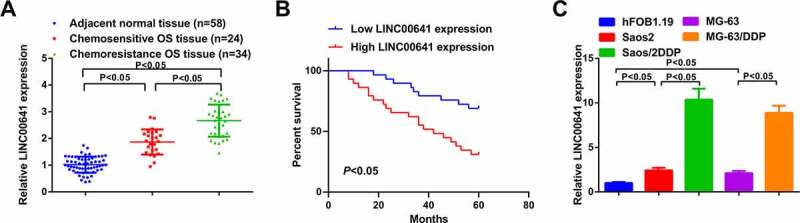
LINC00641 is augmented in DDP-resistant OS tissues and cell lines, and its elevation heralds unpleasing prognosis. (a) RT-qPCR to detect LINC00641 in normal tissues and DDP-sensitive or drug-resistant cancer tissues; (b) Kaplan-Meier method to analyze the function of LINC00641 in OS prognosis; (c) RT-qPCR to examine LINC00641 in hFOB1.19 cells, OS cells and OS DDP-resistant cell lines. *P < 0.05.

The culture of OS cell lines Saos2 and MG-63 (all American Type Culture Collection, Manassas, VA, USA) was exerted in elevated concentration of DDP (Sigma, St.Louis, MO, USA) for more than 6 months, and then DDP-resistant cell lines Saos2/DDP and MG-63/DDP were constructed (Attached Figure 1a). Normal cervical epithelial cell line HaCaT (Shanghai Institute of Biochemistry and Cell Biology, Shanghai, China) was applied. Then all the cells were cultivated in Dulbecco’s Modified Eagle Medium (DMEM, Gibco, Carlsad, CA, CA) comprising 10% fetal bovine serum (FBS) (Gibco), 100 U/mL penicillin and 100 U/mL streptomycin (Sigma Aldrich, Shanghai, China).

Synthesis of shRNA short hairpin RNA targeting LINC00641 (sh-LINC00641) and shRNA interference control (sh-NC) contraposing LINC00641, pcDNA and pcDNA-MCL1 elevated vector (MCL1) was via Genepharma (Shanghai, China). MiR-320d mimic (miR-320d), mimic negative control (miR-NC), miR-320d inhibitor (anti-miR-320d) and inhibitor NC (anti-NC) (all RiboBio Co., Ltd., Guangzhou, China) were put into practice. When the cell concentration of Saos-2/DDP and MG-63/DDP attained 50 ~ 60%, TE transfection of miRNA mimic (10 nM) or vector was adopted via LipofectamineSaos2/DDP 2000 reagent (Carlsbad, California, USA). Ten cells were gathered for 48 h for subsequent analysis [24].

### 2.3 Quantitative real time polymerase chain reaction (RT-qPCR)

Trizol reagent (Invitgen) was utilized to lyse treated cells and extract the total RNA. Reverse transcription into complementary DNA (cDNA) was via reverse transcription kit (Invitgen). RT-qPCR was implemented on the applied biological system 7300 (Invitgen) via SYBR Green RT-PCR kit (Invitgen). Detection of RNA expression was conducted. The calculation of relative expression amount of indicator genes was via 2^−ΔΔCT^ method, and glyceraldehyde-3-phosphate dehydrogenase (GAPDH)/U6 was adopted as standardized internal references [25].

### 2.4 Cell viability assay

The 3-(4,5-dimethylthiazol-2-yl)-2,5-diphenyltetrazolium bromide (MTT) assay was utilized to examine cell viability. Next, the seeding of transfected DDP-resistant cells was into 96-well plates at a density of 5 × 10^3^ cells/well, and incubation together with DDP was conducted with different doses. In different time points (24, 48, 72 h), addition of 20 μL thiazole blue solution was to each well, and then addition of formaldehyde generated via dimethyl sulfoxide dissolving was manifested. At last, the measurement of absorbance was exerted at 490 nm via a microplate reader (Bio-Rad, Hercules, CA, USA).

### 2.5 Cell migration and invasion assay

After the detection of migration and invasion abilities *in vitro* of DDP-resistant cells, the seeding of transfected DDP-resistant cells was utilized into a 24-well plate upper chamber (Becton-Dickinson, Franklin Lake, New Jersey, USA) with or without matrix, and then the culture in serum-free DMEM was carried out. Then the lower chamber in each well was added with complete culture solution comprising 10% FBS, which was regarded as a chemical inducer. After incubation, inactive cells on the upper surface were removed from with a dry cotton swab. Then fixation of the cells was conducted via methanol with ventilating appropriately the chamber. Next, after staining with 0.1% crystal violet, non-transferring cells on the upper layer were lightly wiped off with a cotton swab, and were immediately observed in five visual fields under a phosphate buffer saline (PBS) 400-fold microscope for counting [26].

### 2.6 Cell apoptosis analysis

After transfection, analysis of the apoptosis rate of DDP-resistant cells was via Annexin V- fluoresceinisothiocyanat/ propidium iodide apoptosis detection kit (Solarbio, Beijing). All specimens were in triplicate [10].

### 2.7 Total RNA extraction and quantitative reverse transcriptase polymerase chain (RT-qPCR)

With extraction of total RNA, TRIzol reagent was obtained from Invitrogen Company, and applied following the manufacturer’s instructions. After cDNA synthesis, Power SYBR Green (TaKaRa, Shiga, Japan) was applied on ABI Step-One real-time system (Thermo Fisher, Waltham, MA, USA) for performing qPCR. The relative quantitative method (2^−ΔΔCT^) was used to calculate gene expression amount. U6 and GAPDH were applied as normalized genes. The primer sequences were listed in Table 1.

**Table 1.** Primer sequences

Genes	Primer sequences (5’–3’)
LINC00641	Forward: 5’–GTAACTCTATGTACAA CGTTAA-3’
	Reverse: 5’-TAGAAGTCAACTCATTATGCTGCTG-3’
MiR-320d	Forward: 5’-AAAAGCTGGGTTGAGAGGA-3’
	Reverse: 5’-TCCTCTCAACCCAGCTTTT-3’
MCL1	Forward: 5’-GGGCAGGATTGTGACTCTCATT-3’
	Reverse: 5’-GATGCAGCTTTCTTGGTTTATGG-3’
Bax	Forward: 5’-GGATCGAGCAGAGAGGATGG-3’
	Reverse: 5’-TGGTGAGTGAGGCAGTGAGG-3’
Bcl-2	Forward: 5’-CTGGTGGACAACATCGCTCTG-3’
	Reverse: 5’-GGTCTGCTGACCTCACTTGTG-3’
U6	Forward: 5’-GCTTCGGCAGCACATATACTAA-3’
	Reverse: 5’-AACGCTTCACGAATTTGCGT-3’
GAPDH	Forward: 5’-AAGGTGAAGGTCGGAGTCAA-3’
	Reverse: 5’-AATGAAGGGGTCATTGATGG-3’

### 2.8 Western blot assay

The extraction of cell protein was conducted from 6-well plate via Radioimmunoprecipitation Assay lysis buffer solution consisting of protease inhibitor. After the quantification via bicinchoninic acid kit (Thermo Fisher Science), the cell protein was diluted to identical concentration in loading buffer and denatured at 95°C, then the electroblot of 20 μg protein was onto Polyvinylidene fluoride membrane via sodium dodecyl sulfate polyacrylamide gel electrophoresis. After seal with skim milk, the protein was co-cultured separately with anti- MCL1 (1: 1000, 5453), caspase-3 (1: 1000; 9661), PARP (1: 1000; 9542) and GAPDH (1: 1000, 2118), and all antibodies were from Cell Signaling Technology. Then the detection of the protein was utilized via chemiluminescence detection system (GE Healthcare, Chicago, IL, USA) [27].

### 2.9 The luciferase activity assay

The cloning of LINC00641 mRNA consisting of miR-320d wild-type (WT) and mutant (MUT) binding sequences was separately exerted into psiCHECK™-2 luciferase plasmid (Promega, Shanghai, China). Saos2/DDP and MG-63/DDP cells were seeded in a 24-well plate, and then respectively co-transfected with miR-320d mimic/miR-NC and LINC00641-WT/MUT. Finally, analysis of the luciferase activity was carried out via dual luciferase assay kit (Promega) [11].

### 2.10 RNA pull-down assay

The association between LINC00641 and miR-320d was evaluated by biotinylated RNA pull down assay. Biotin-labeled miR-361-3p probes and NC probes were provided by GenePharma. In short, 1 × 10^5^ HOS cells were obtained, then lysed and treated with ultrasound. Probes were incubated at 25°C for 3 h using C-1 magnetic beads (Life Technologies). The cell lysates with miR-361-3p or oligo probes were incubated at 4°C for 24 h and then washed with PBS buffer. Binding RNA was purified with Trizol reagent and RT-qPCR was performed [28].

### 2.11 RNA immunoprecipitation (RIP) assay

RIP assay was conducted using human Argonaute2 (Ago2) antibody (anti-Ago2) with the help of the Magna RIP Kit (EMD Millipore, Billerica, MA, USA). Magnetic beads were incubated with indicated antibodies. The cells were lysed in RIP lysis buffer. Cell lysates were obtained and magnetic beads were combined to incubate with indicated antibodies. RNA was purified from the precipitate and analyzed by RT-qPCR [29].

### 2.12 Tumor xenograft test

Lentivirus vector (GenePharma, Shanghai, China), that is lentit-short hairpin LINC00641, named sh-LINC00641, was adopted to facilitate stabilization of LINC00641, and the control of messy code of shRNA (sh-NC) was put into use. Meanwhile, 6-week-old male Balb/c mice (five in each group) were collected from National Laboratory Animal Center (Beijing, China). This study had obtained ratification from Animal Research Committee, Huai’an Second People’s Hospital. All animal studies were executed on the grounds of Animal Research: Reporting of *In Vivo* Experiments Guidelines and the Basel Declaration. All animals were taken humane care in line with the guidelines of the National Institutes of Health. After steady transfection with sh-LINC00641 or sh-NC, subcutaneous injection of the cells (5 × 10^6^) was utilized into the left abdomen of nude mice. After 7-d injection, the mice were given PBS (Invitrogen) or 3 mg/kg DDP (DDP) (Sigma) and their tumor volume was measured weekly. All mice were implemented euthanasia after 28 d, and their resected tumors were weighed (Attached Figure 1b).

### 2.13 Terminal deoxynucleotidyl transferase-mediated dUTP-biotin nick end labeling (TUNEL)

Xenografted tumor sections were dewaxed with xylene and rehydrated with gradient alcohol. The 4% paraformaldehyde was applied for fixing the sections. Then the sections were treated with proteinase K solution (20 μg/mL) and TUNEL reaction buffer. Then the sections were utilized staining with diaminobenzidine and hematoxylin. Next the sections were dehydrated with gradient ethanol, treated with xylene, and sealed with neutral resin. The observation of TUNEL positive cells (brown granules) was conducted under the microscope.

### 2.14 Statistical analysis

All statistical analysis was adopted via GraphPad Prism 7(GraphPad Inc, San Diego, California, USA). Manifestation of the data was as mean ± standard deviation. The pertinence among LINC00641, miR-320d and MCL1 was analyzed via Pearson test. Kaplan-Meier survival curve was drawn and logarithmic rank test were put into use to analyze the survival distinctions between two groups. Student’s *t*-test or one-way analysis of variance (ANOVA) was exerted to evaluate the prominent distinctions between groups. *P* < 0.05 was considered the apparent difference.

## 3 Results

Here, it was aimed to investigate the role of LINC00641 and its downstream molecular mechanisms in OS cells. A series of *in vitro* experiments was conducted and it was found that LINC00641 regulated cell proliferation, invasion and apoptosis, and promoted DDP resistance in OS cells through miR-320d/MCL1 axis. Therefore, the function and mechanism of LINC00641 in OS were first investigated in the data, providing new insights into the pathogenesis of OS.

### 3.1 LINC00641 manifests elevation in DDP-resistant OS tissues and cancer cell lines, which portends unpleasing prognosis

For the sake of exploring the function of LINC00641 in DDP-resistant OS, detection of LINC00641 was conducted in DDP-sensitive and -resistant OS tissues and cells. The results clarified that LINC00641 was saliently augmented in OS tissues and cells, especially in DDP-resistant tumors and cell lines (Saos2/DDP and MG-63/DDP) (Figure 1a and b). Clinical correlation analysis manifested higher LINC00641 level was associated with higher Enneking stage, larger tumor size, and positive distant metastasis in patients with OS (*P* < 0.05, Table 2). Additionally, 58 patients who had been under DDP therapy were executed 60-month follow-up. And it came out that the overall survival rate of patients with ascending LINC00641 was inferior to that of patients with depressive one (Figure 1c), which insinuated that the augmentation of LINC00641 was a predictor of unpleasing prognosis of OS patients.

**Table 2.** The relationship between LINC00641 expression level and clinical characteristics in patients with OS

Characteristics	Patients (n = 58)	LINC00641 expression		*P*
		Low (n = 29)	High (n = 29)	
**Age (years)**				
<18	39	21	18	0.576
≥18	19	8	11	
**Gender**				
Male	33	18	15	0.596
Female	25	11	14	
**Pathological**				
G1	10	6	4	0.182
G2	19	12	7	
G3-G4	29	11	18	
**Enneking stage**				
I–IIA	26	17	9	0.035*
IIB-III	32	12	20	
**Tumor size**				
<8 cm	35	22	13	0.016*
≥8 cm	23	7	16	
**Distant metastasis**				
Yes	18	5	13	0.023*
No	40	24	16	

### 3.2 Inhibition of LINC00641 restrains the proliferation, migration and invasion, but promotes apoptosis of OS cells

**Figure 2.**
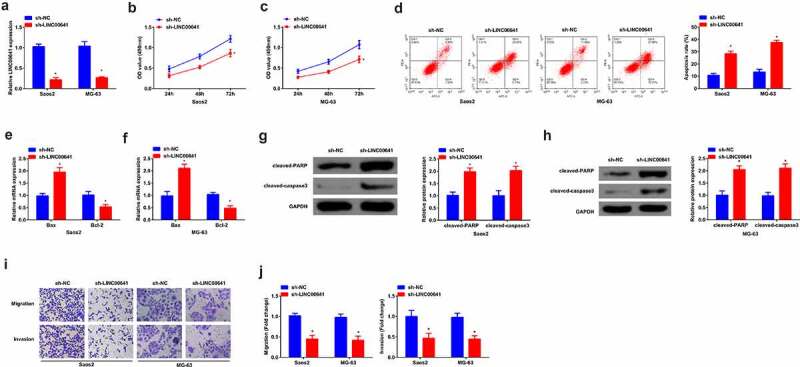
Inhibition of LINC00641 represses the proliferation, migration and invasion, but promotes apoptosis of OS cells. (a) qPCR to detect the expression of LINC00641 in Saos2 and MG-63 cells; (B/C) MTT to detect the proliferation ability of Saos2 and MG-63 cells; (d) Flow cytometry to detect apoptosis of Saos2 and MG-63 cells; (E/F) qPCR to detect the mRNA expression of Bax and Bcl-2 in Saos2 and MG-63 cells. (G/H) Western blot to detect the protein expression of Caspase-3 and PARP in Saos2 and MG-63 cells; (I/J) Transwell to detect the migration and invasion of Saos2 and MG-63 cells. * vs. the sh-NC, *P* < 0.05.

To investigate the role of LINC00641 in OS, LINC00641 was first down-regulated in two parent strains Saos2 and MG-63 to explore its role. Saos2 and MG-63 were transfected with sh-LINC00641 and its NC, and via qPCR was detected that the expression of LINC00641 was clearly decreased in cells (Figure 2a). Via MTT assay was detected cell proliferation, and it was found that the proliferation ability of Saos2 and MG-63 was apparently reduced after LINC00641 was inhibited (Figure 2b and c). Apoptosis level was detected by flow cytometry, and it was found that repression of LINC00641 promoted cell apoptosis (Figure 2d). In addition, apoptosis-related proteins Bax, Bcl-2, Cleaved caspase3 and PARP expression levels were detected by qPCR or Western blot, and the results manifested Bax, cleaved-PARP, cleaved-caspase3 expression was elevated, while Bcl-2 expression was decreased clearly after LINC00641 down-regulation (Figure 2e-h). Transwell was applied to detect the migration and invasion abilities of cells and it was found that low expression of LINC00641 effectively restrained the migration and invasion of Saos2 and MG-63 cells (Figure 2i-j). In summary, it was found that inhibition of LINC00641 constrained the proliferation, migration and invasion, but promoted apoptosis of OS cells.

### 3.3 Knock-down LINC00641 attenuates progression of DDP-resistant OS cells

**Figure 3.**
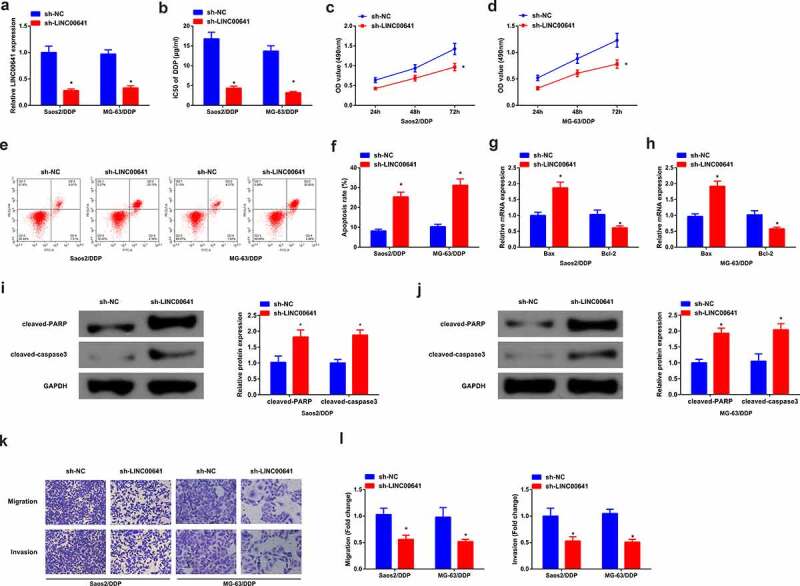
Knock-down of LINC00641 gene is able to restrain the survival and advancement of DDP-resistant OS cells. (a) After transfection of sh-LINC00641, detection of LINC00641; (b) IC50 values; (c-d) MTT assay to detect the survival rate and cell proliferation; (e-f) Flow cytometry to analyze the apoptosis rate; (g-h) RT-qPCR to examine Bcl-2 and Bax mRNA; (i-j) Western blotto detect caspase-3 and PARP protein expression; (k-l) Transwell method to analyze the migration and invasion abilities. In Saos2/DDP and MG-63/DDP cells. * vs. the sh-NC, *P* < 0.05.

In order to further investigate the biological effect of LINC00641 on DDP-resistant OS cells, sh-NC/LINC00641 was transfected into Saos2/DDP and MG-63/DDP cells, with validating the transfection efficiency. The results manifested that LINC00641 was declined in cells transfected with sh-LINC00641 (Figure 3a). After 48 h of DDP treatment with different concentrations, it was found that LINC00641 loss reduced the IC50 value of Saos2/DDP and MG-63/DDP cells, and repressed the proliferation ability of DDP resistant cells (Figure 3b and d). In addition, flow cytometry results showed that LINC00641 knockdown clearly induced apoptosis of drug-resistant cells (Figure 3e and f). Meanwhile, qPCR results manifested LINC00641 deletion promoted the expression of Bax in Saos2/DDP and MG-63/DDP cells, while restrained the expression of Bcl-2, further suggesting that LINC00641 deletion could promote apoptosis of drug-resistant cells (Figure 3g and h). Moreover, cleaved-PARP, cleaved-caspase3 results of Western blot detection showed that LINC00641 inhibition clearly increased cleaved-PARP, cleaved-caspase3 protein (Figure 3i and j). Transwell assay was then performed, and the results showed that LINC00641 knockdown could repress the migration and invasion abilities of Saos2/DDP and MG-63/DDP cells (Figure 3k and l). In conclusion, knockdown of LINC00641 reduced the resistance of DDP resistant cells to DDP by restraining the proliferation and inducing apoptosis of DDP resistant OS cells.

### 3.4 LINC00641 emerges direct binding and negative mediation to miR-320d

**Figure 4.**
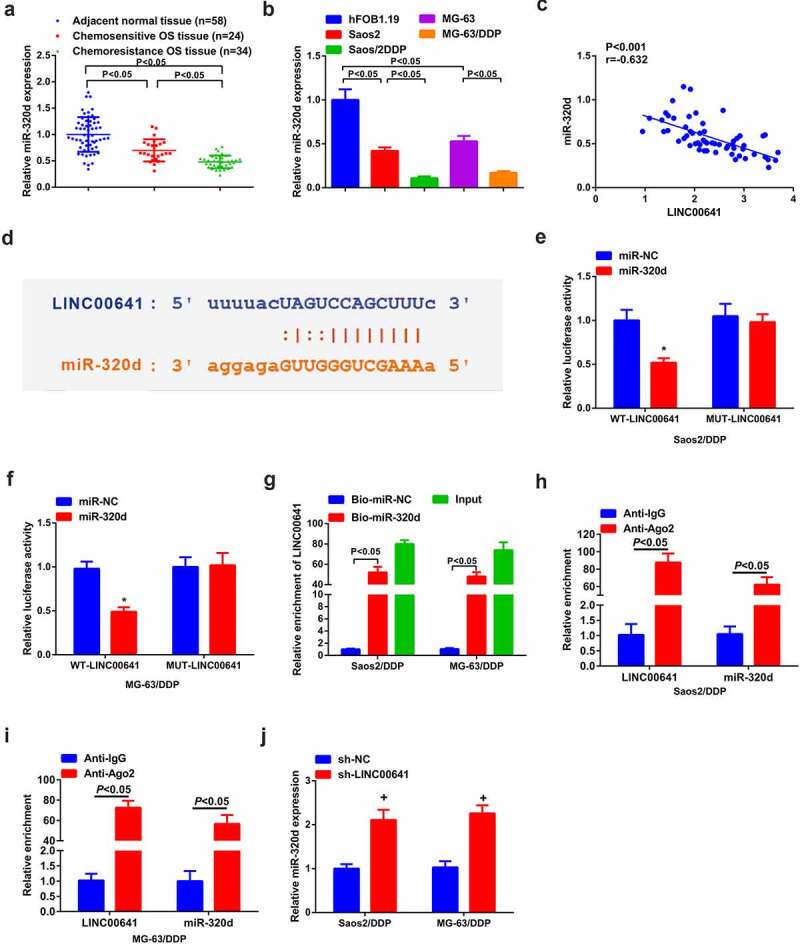
LINC00641 directly binds to miR-320d to negatively control it. (a-b) RT-qPCR to detect miR-320d in DDP-sensitive or drug-resistant cancer tissues and cell lines; (c) The analysis of the pertinence of LINC00641 with miR-320d; (d) The binding sequence of LINC00641 and miR-320d was predicted; (e-f) LINC00641-WT/MUT or miR-320d mimic/miRNC co-transfected into Saos2/DDP and MG-63/DDP cells for the luciferase activity detection; (g) RT-qPCR to examine LINC00641 after biotinylated miR-320d/NC; (H/I) RIP to detect the enrichment of LINC00641 or miR-320d in anti-ago2 combination precipitation. (j) RT-qPCR to detect miR-320d in Saos2/DDP and MG-63/DDP cells transfected with sh-NC/LINC00641. *P < 0.05.

On the grounds of the detection of miR-320d, it was observed that miR-320d was silenced in OS tissues and cell lines in comparison with normal one, especially in DDP-resistant tumors and cell lines (Figure 4a and b). Then, miR-320d and LINC00641 were negatively absolutely correlated in OS patients (Figure 4c). The above results suggested that miR-320d was likely to be relevant to the resistance of LINC00641 toward DDP. In order to elaborate this hypothesis, bioinformatics was conducted to analyze and prefigure that miR-320d was the target of LINC00641 and they had hypothetic binding site (Figure 4d). Meanwhile, it was clear that transfection of miR-320d mimic reduced the luciferase activity of WT-LINC00641 reporter vector, but could not reduce that of empty vector or MUT-LINC00641 reporter vector in Saos2/DDP and MG-63/DDP cells (Figure 4e and f). Meanwhile, the further verification was gained that a direct reciprocity of miR-320d with LINC00641 existed, since noteworthy enrichment of LINC00641 was examined in both Saos2/DDP and MG-63/DDP cells (Figure 4g). RIP test found that LINC00641 and miR-320d were significantly enriched in the anti-ago2 group compared with the anti-igg group (Figure 4h and i). Furthermore, it was discovered that deletion of LINC00641 accelerated miR-320d in Saos2/DDP and MG-63/DDP cells (Figure 4j). Therefore, LINC00641 was able to negatively modulate miR-320d.

### 3.5 Knock-down of LINC00641 gene represses DDP-resistance of DDP-resistant OS cells via modulating miR-320d

**Figure 5.**
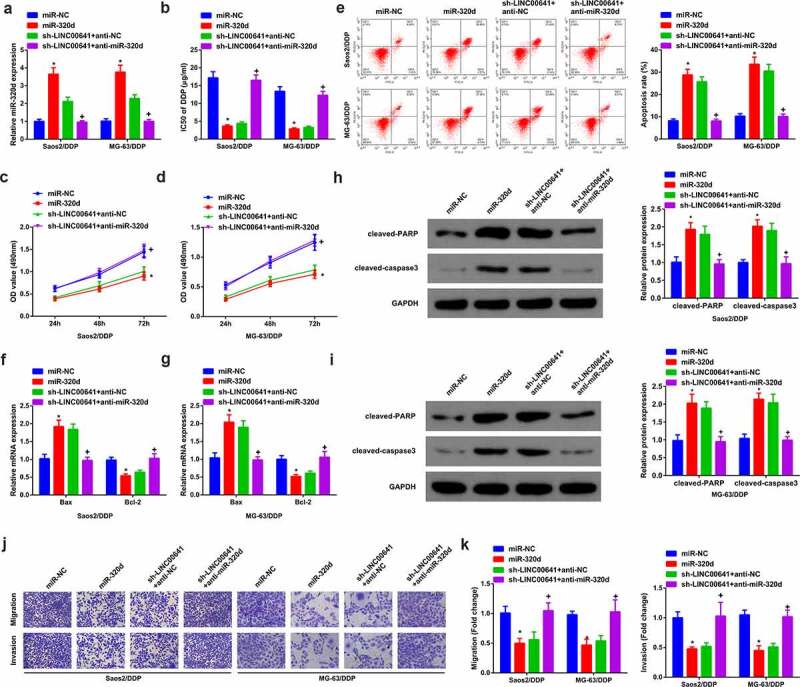
Knock-down LINC00641 is capable of reducing DDP-resistance of DDP-resistant OS cells via mediating miR-320d. (a) qPCR to detect miR-320d in cells; (b) IC50 value of cells in each group; (c-d) MTT assay to detect the survival rate and proliferation; (e) Flow cytometry to analyze apoptosis rates; (f-g) qPCR to examine Bcl-2 and Bax mRNA; (h-i) Western blot to detect caspase-3 and PARP protein expression; (j-k) Transwell method to analyze the migration and invasion abilities. In C-I, in Saos2/DDP and MG-63/DDP cells. * vs. the miR-NC, *P* < 0.05; + vs. the sh-LINC00641 + anti-NC, *P* < 0.05.

Saos2/DDP and MG-63/DDP cells were transfected with miR-NC/320d, sh-NC/LINC00641 or sh-LINC00641 + anti-miR-NC/miR-320d to explore whether miR-320d participated in drug resistance of LINC00641 (Figure 5a). After transfection, the progression of DDP-resistant cells was observed. The results showed that overexpression of miR-320d inhibited the IC50 value (Figure 5b), proliferation (Figure 5c and d), migration (Figure 5j) and invasion (Figure 5k), but promoted apoptosis of DDP drug-resistant cells (figure 5f–i). In addition, low expression of miR-320d reversed the therapeutic effect of repressing LINC00641 on DDP resistant cells, significantly increasing IC50 value (Figure 5b), proliferation (Figure 5c and d), migration (Figure 5j), invasion (Figure 5k) and clearly decreasing apoptosis (figure 5f–i). As has been noted, it became clear that knock-down of LINC00641 was capable of restraining DDP-resistance of DDP-resistant OS cells via targeting miR-320d.

### 3.6 MCL1 is the alternative target of miR-320d

**Figure 6.**
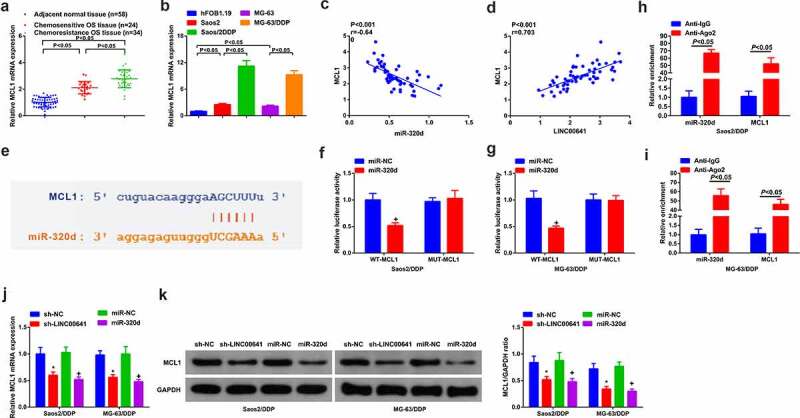
MCL1 is the alternative target of miR-320d. (a) RT-qPCR to detect MCL1 in normal tissues and DDP-sensitive or drug-resistant cancer tissues; (b) RT-qPCR to examine MCL1 in hFOB1.19 cells, OS cells and OS DDP-resistant cell lines; (c-d) Pearson test to analyze the pertinence of MCL1, LINC00641 with miR-320d; (e) The binding site of MCL1 with miR-320d predicted via bioinformatics website; (f-g) The luciferase activity assay to testify the targeting of MCL1 with miR-320d; (h-i) RIP to detect the enrichment of LINC00641 or miR-320d in anti-ago2 combination precipitation; J-K. qPCR and Western blot to detect MCL1. * vs. the sh-NC, *P* < 0.05; + vs. the miR-NC, *P* < 0.05.

Detection of MCL1 was in DDP-sensitive and -resistant OS tissues and cells. The results clarified that MCL1 was saliently elevated in OS tissues and cells, especially in DDP-resistant tumors and cell lines (Saos2/DDP and MG-63/DDP) (Figure 6a and b). Moreover, it was found that clinical data manifested miR-320d was negatively associated with MCL1, while LINC00641 was positively linked with MCL1 (Figure 6c and d). Therefore, it was presumed that a certain pertinence of MCL1 with miR-320d existed. Then starBase v3.0 was exerted to predict underlying targets of miR-320d. The results illustrated that MCL1 3ʹuntranslated region (UTR) and miR-320d had complementary binding sites (Figure 6e). In MG63/DDP and Saos-2/DDP cells transfected with miR-320d, the luciferase activity of MCL1 3ʹUTR-WT reporter gene emerged acute declination, while in any group that of MCL1 3ʹUTR-MUT reporter gene did not present conspicuous fluctuation (figure 6f and g). RIP test found that miR-320d and MCL1 were significantly enriched in the anti-ago2 group compared with the anti-igg group (Figure 6h and i).Additionally, MCL1 were dramatically silenced in MG63/DDP and Saos-2/DDP cells transfected with sh-LINC00641 or miR-320d (Figure 6j and k). The above results concluded that miR-320d is capable of interacting with MCL1, to attenuate MCL1 in MG63/DDP and Saos-2/DDP cells.

### 3.7 Refrained LINC00641 gene antagonizes DDP-resistance of DDP-resistant OS cells via modulating MCL1

**Figure 7.**
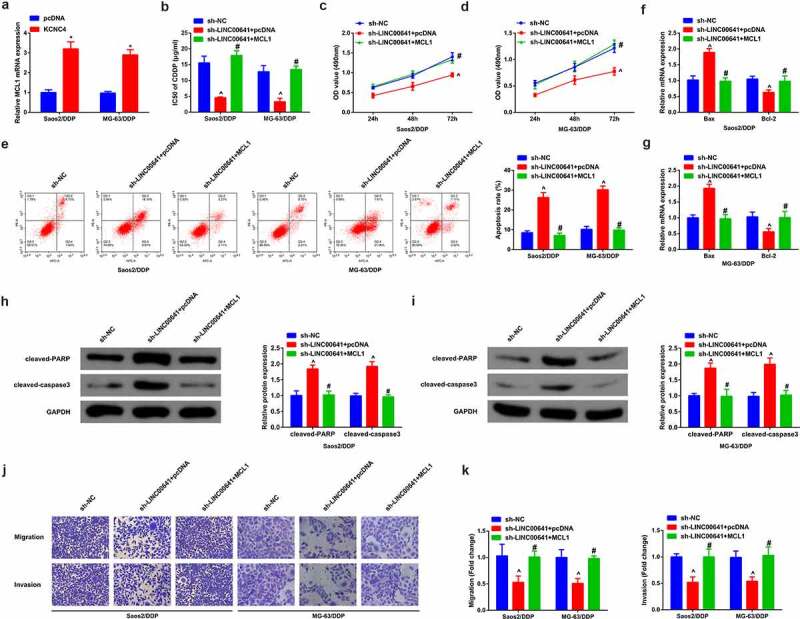
Restrained LINC00641 gene emerges antagonism toward DDP resistance of DDP-resistant OS cells via controlling MCL1. (a) RT-qPCR to detect MCL1; (b) IC50 value of cells in each group; (c-d) MTT assay to examine the proliferation ability; (e) Flow cytometry to detect apoptosis rates; (f-g) RT-qPCR to examine Bcl-2 and Bax mRNA; (h-i) Western blot to detect caspase-3 and PARP protein expression; (j-k) Transwell method to analyze the migration and invasion abilities. Apart from B, in Saos2/DDP and MG-63/DDP cells. * vs. the pcDNA, *P* < 0.05; # vs. the sh-LINC00641 + pcDNA, *P* < 0.05.

For the sake of verifying whether MCL1 was implicated in DDP-resistance triggered via LINC00641, sh-NC/LINC00641 and sh-LINC00641 + MCL1/pcDNA were transfected into Saos2/DDP and MG-63/DDP cells. After validating transfection efficiency, the results uncovered that MCL1 was ascending in cells transfected with MCL1 (Figure 7a). Examination of the advancement of DDP-resistant cells was conducted. As a matter of reality, the results indicated that transfection of MCL1 was able to weaken repressive effect in the progression of Saos2/DDP and MG-63/DDP cells triggered via deletion of LINC00641, repressing IC50 value (Figure 7b), proliferation (Figure 7c,d), migration (Figure 7j), invasion (Figure 7k), but promoting apoptosis (figure 7f-i).It was validated that knockdown of LINC00641 gene was inclined to emerge antagonism toward drug resistance of DDP-resistant OS cells via the mediation of MCL1. Besides, on the basis of the above results, knock-down of LINC00641 gene tended to block MCL1 via emulative interaction with miR-320d, thus expediting the sensitivity of OS toward DDP.

### 3.8 Depressive LINC00641 constrains OS tumor and enhances DDP sensitive growth

**Figure 8.**
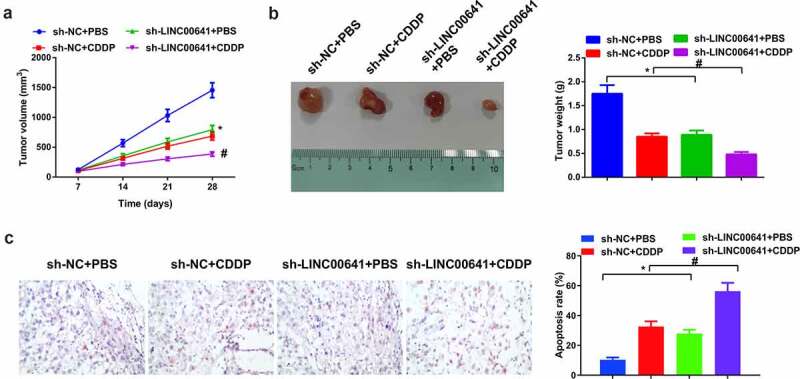
Depressive LINC00641 suppresses OS tumor and escalates development of DDP sensitivity. (a) Tumor volume of mice in each group; (b) tumor images and weight variations of mice in each group; (c) TUNEL staining to examine apoptotic cells. * vs. the sh-NC + PBS, *P* < 0.05; # vs. the sh-LINC00641 + PBS, *P* < 0.05.

Next, OS mice xenograft tumor model was established *in vivo* to further figure out the impact of LINC00641 deletion on tumor growth. As shown in (Figure 8a and b), DDP therapy or declination of LINC00641 gave rise to a decrease in tumor volume and weight, which indicated that DDP therapy or silenced LINC00641 repressed the growth of OS tumor *in vivo*. In the meantime, repressive effect on tumor growth was manifested more observably via the combination of sh-LINC00641 with DDP therapy. Apart from that, the apoptosis of tumor tissues was detected and it displayed that DDP therapy or silenced LINC00641 boosted the apoptosis of cells. The effect of boosting cell apoptosis was exhibited more signally via the combination of sh-LINC00641 with DDP (Figure 8c), which suggested that deletion of LINC00641 was able to facilitate anti-tumor effect of DDP *in vivo*.

## 4 Discussion

DDP has been identified as the pivotal medicine for cancer chemotherapy since its discovery [30], has been widely used in the treatment of various solid tumors. It is a metal-containing drug, which does not make it cross-resistant to other drugs in some key pathways [31]. Therefore, only elucidating the mechanism of OS resistance to DDP has important therapeutic value. The purpose of this study was to investigate the action mechanism of LINC00641 in DDP resistance in OS. The results showed that LINC00641 knockdown reduced DDP resistance of Saos2/DDP and MG-63/DDP cells, repressed cell proliferation, migration and invasion, but promoted apoptosis through miR-320d/MCL1 axis.

In recent years, a great deal of studies hasve verified the pertinence of maladjustment of lncRNAs with DDP-resistance in tumors involving cancer, and investigated numerous lncRNAs linked with DDP-resistance. For instance, ROR is implicated in OS resistance toward DDP via miR-153-3p/ABCB1 axis and unlocks novel mediative axis of OS cells toward DDP, which is likely to provide therapeutic targets for OS patients [32]; and the study of LINC00641 in OS and its influences on OS prognosis and DDP resistance have remained still obscure. In this study, it was found that LINC00641 was elevated in OS tissues and cells. At the same time, LINC00641 was dramatically augmented in DDP-resistant tumors and cell lines, insinuating that LINC00641 took on a latent regulatory effect in DDP-resistance of OS. On this basis, advancement of DDP-resistant OS cells was detected, and it was discovered that knock-down of LINC00641 gene was able to restrain advancement and metastasis of DDP-resistant cells thus weakening their DDP resistance. In the meantime, steadily transfected MG-63/DDP cells were utilized to construct xenografted tumor model in OS mice. The results manifested that DDP therapy or silenced LINC00641 contributed to a declination in tumor volume and weight, which signified that DDP therapy or depressive LINC00641 attenuated the development of OS tumor *in vivo*; Meanwhile, the combination of sh-LINC00641 and DDP had a more remarkable repressive effect on tumor growth, which was consistent with the experimental results *in vitro*. Taking the above biological behavior of LINC00641 into consideration, the pertinence of LINC00641 with overall survival period was evaluated, and it was determined that ascending LINC00641 was capable of pointing out the unpleasing prognostic of OS patients.

It is reported that lncRNA is utilized as competitive endogenous RNA (ceRNA) of miRNAs, and to modulate gene indirectly in miscellaneous biological processes involving cancer [33]. Therefore, it was guessed that whether LINC00641 performed as a ceRNA to mediate miR-320d/MCL1 axis and taking on effect in stimulating cancer in OS. In line with bioinformatics prediction program, miR-320d manifested as the target of LINC00641, and an immediate binding of LINC00641 to miR-320d was verified. Then, the analysis of miR-320d was conducted and it was observed that miR-320d was silenced in OS tissues and cell lines. In OS patients, miR-320d and LINC00641 were immediately validated to be completely negatively correlated.

Studies have elucidated members of miR-320 family have been reported to be implicated in multiple malignant tumors, including glioma [34], colon cancer [35], large B-cell lymphoma [36]. Nevertheless, the research on the effect and mechanism of miR-320d in the development of OS and its DDP-resistance has remained still scarce. For the sake of verifying whether miR-320d was relevant to DDP-resistance incurred via LINC00641, augmentation of miR-320d and rescue experiments were carried out. The results uncovered that miR-320d dampened the activity and advancement of DDP-resistant OS cells, while transfection of miR-320d inhibitor could restore the repressive effect of DDP-resistance of DDP-resistant OS cells triggered via deletion of LINC00641. Prior to this, LINC00641 ameliorated DDP-resistance of DDP-resistant OS cells via targeting miR-320d.

Many evidences have expounded that MCL1 also participates in tumor development and is under the mediation of miRNA. For instance, miR-153-3p modulates development of ovarian cancer *in vivo* and *in vitro* via targeting MCL1 gene [37]. In this study, it was found that MCL1 was signally augmented in OS tissues and cells and was targeted via miR-320d. Moreover, it was observed via transfection of elevated MLC1 plasmid into cells with suppressive LINC00641 that enhancive MLC1 could reverse the impact of LINC00641 deletion on DDP-resistant OS cells, indicating that knock-down of LINC00641 gene was inclined to antagonize resistance of DDP-resistant OS cells via controlling MCL1.

Nevertheless, there are still some limitations in this study. *In vivo* experiments were worth conducting to further explore the function of miR-320d/MCL1, the downstream gene of LINC00641, in OS DDP-resistance. Besides, limited patient specimens might not completely validate accuracy of the results.

## 5 Conclusion

In a word, the study illustrates that LINC00641 is elevated in OS tissues and cells, and its augmentation is connected with unpleasing prognosis of OS patients. Moreover, declining LINC00641 could restrain the activity of OS cells, and further mechanism study uncovers that LINC00641 principally took on effect in modulating miR-320d/MCL1 axis. The discoveries suggest that LINC00641 is likely to be a novel molecular marker and therapeutic target of OS. To sum up, this study testifies that LINC00641 facilitates the advancement and metastasis of OS cells via negatively modulating miR-320d/MCL1, thus expediting the occurrence and development of OS.

## Supplementary Material

Supplemental Material
