# Supplementary figures and images for: Long non-coding RNA long intergenic non-coding 00641 mediates cell progression with stimulating cisplatin-resistance in osteosarcoma cells via microRNA-320d/myeloid cell leukemia-1 axis
Source: Bioengineered. 2022 Mar 10;13(3):7238–52. doi: 10.1080/21655979.2022.2045090 (PMC9208475; doi:10.1080/21655979.2022.2045090)

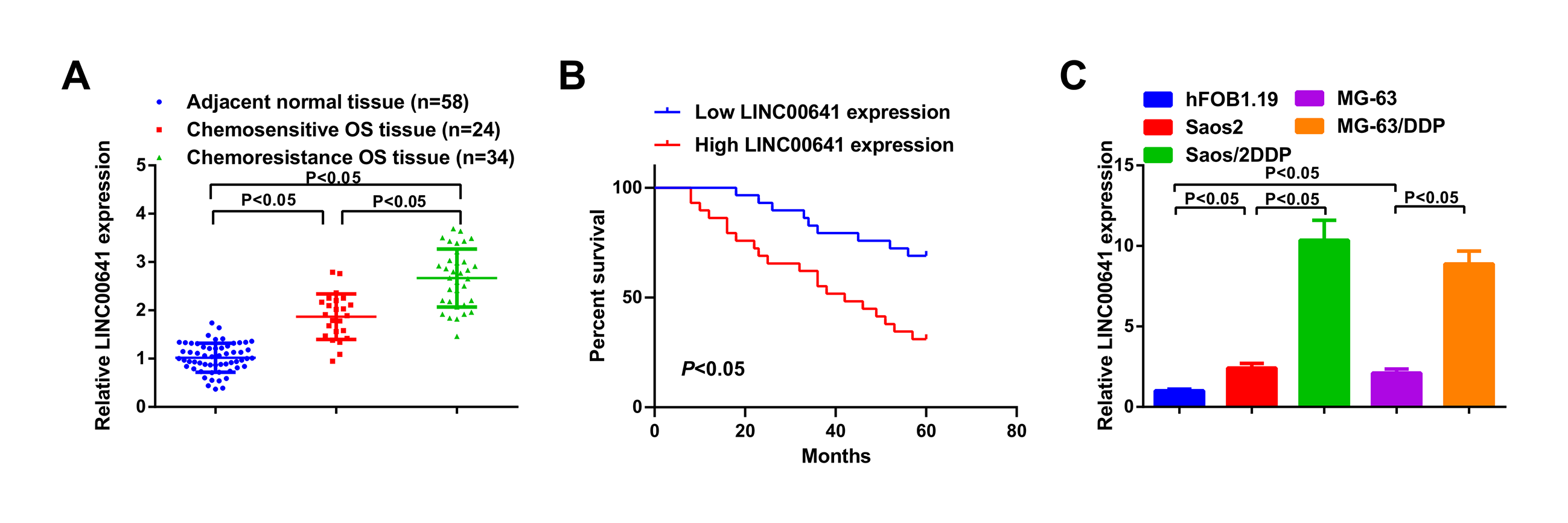

Supplement: Supplemental Material [file KBIE_A_2045090_SM9594.zip › supplementary/Attached Figure1.tif]
